# Clinical insights into hematologic malignancies and comparative analysis of molecular signatures of acute myeloid leukemia in different ethnicities using an artificial intelligence offering

**DOI:** 10.1097/MD.0000000000027969

**Published:** 2021-12-23

**Authors:** Jane L. Snowdon, Dilhan Weeraratne, Hu Huang, David Brotman, Shang Xue, Van C. Willis, Young Kyung Lee, Kibum Jeon, Dae Young Zang, Hyo Jung Kim, Ho Young Kim, Boram Han, Miyoung Kim

**Affiliations:** a IBM Watson Health, Cambridge, MA, USA; b Department of Laboratory Medicine, Hallym University Sacred Heart Hospital, Anyang, Republic of Korea; c Department of Laboratory Medicine, Hallym University Hangang Sacred Heart Hospital, Seoul, Republic of Korea; d Department of Internal Medicine, Hallym University Sacred Heart Hospital, Anyang, Republic of Korea; e Department of Laboratory Medicine, Asan Medical Center, University of Ulsan College of Medicine, Seoul, Republic of Korea.

**Keywords:** cancer genetics, hematology, leukemia, molecular aspects

## Abstract

Next generation sequencing generates copious amounts of genomics data, causing manual interpretation to be laborious and non-scalable while remaining subjective (even for highly trained specialists). We evaluated the performance of the artificial intelligence-based offering Watson for Genomics (WfG), a variant interpretation platform, in hematologic malignancies for the first time.

Next generation sequencing was performed for patients treated for various hematological malignancies at Hallym University Sacred Heart Hospital, South Korea, between December 2017 and August 2020 using a 54-gene panel. Both WfG and expert manual curation were used to evaluate the performance of WfG. Acute myeloid leukemia (AML) molecular profiles were compared between Koreans and other ethnic groups using a publicly available dataset.

Seventy-seven patients were analyzed (AML: 45, myeloproliferative neoplasms: 12, multiple myeloma: 7, myelodysplastic syndromes: 6, and others: 7). The concordance between the manual and WfG interpretations of 35 variants in 11 random patients was 94%. Among all patients, WfG identified 39 (51%) with at least 1 clinically actionable therapeutic alteration (i.e., a variant targeted by a United States Food and Drug Administration [US FDA]-approved drug, off-label drug, or clinical trial). Moreover, 46% of these patients (18/39) had genes that were targeted by a US FDA-approved therapy. WfG identified diagnostic or prognostic insights in 65% of the patients with no targetable alterations. In those with AML, *FLT3*-internal tandem duplications or tyrosine kinase domain mutations were less frequent among Koreans than among Caucasians (6.7% vs 30.2%, *P* < .001) or Hispanics (6.7% vs 28.3%, *P* = .005), suggesting ethnic differences.

Variant interpretation using WfG correlated well with manually curated expert opinions. WfG provided therapeutic insights (including variant-specific drugs and clinical trials that cannot easily be provided by expert manual curation), as well as diagnostic and/or prognostic information.

## Abstract 2

Supplemental Digital Content is available in the text

## 1 Introduction

The premise of precision oncology is to deliver targeted treatments according to the specific molecular profile of each patient.^[1,2]^ Next-generation sequencing (NGS) has revolutionized precision oncology by providing tumor-specific genomic alteration profiles.^[1–3]^ However, a major challenge is the exponentially increasing medical literature on gene variants; this necessitates the constant updating of on- and off-label drug treatments and clinical trials, thereby overwhelming medical professionals.^[4–6]^ Accurate manual interpretation of large amounts of NGS data is laborious, time-consuming, and non-scalable while remaining subjective – even for highly trained specialists.^[3]^ However, reliable, evidence-based interpretation of genomic variants (i.e., their therapeutic, diagnostic, and prognostic implications) as well as the identification of pertinent targeted drugs and suitable clinical trials are integral to maximizing the clinical benefit toward patients with cancer at the point-of-care.^[3]^

Artificial intelligence-based solutions such as Watson for Genomics (WfG) provide an alternative to the expert manual curation of NGS data, thereby delivering relevant information to the clinician about potential treatments at the optimal time. WfG leverages the clinical lab's workflow and NGS data derived from a patient's tumor to annotate uploaded cases and prioritize mutations. The performance of WfG has been evaluated in different types of solid tumors by the manufacturer as well as by independent NGS specialists.^[3,7]^ In a representative study of 198 patients with different types of malignancies such as breast, gastric, and lung cancers, WfG showed a concordance rate of 89.8% with in-house specialists, covered 84.6% of all targeted therapies that the experts proposed, and offered an additional 225 therapeutic options.^[3]^ However, the performance of WfG in patients with hematologic malignancies has not yet been reported. Nevertheless, it is hypothesized that WfG can identify specific actionable mutations, thereby contributing to improved clinical outcomes and also facilitating the interrogation of racial disparities in cancer-specific molecular profiles. Notably, the majority of published cancer studies involving NGS have focused on Caucasian populations; only a few have compared the molecular profiles of tumors between patients of different ethnic groups.^[8]^

To ascertain the clinical utility of WfG in hematologic malignancies, we investigated its performance when analyzing NGS data from patients with hematologic malignancies in this first-of-its-kind study. WfG interpretations of randomly selected cases were compared to those that were manually curated by experts. We analyzed the performance of WfG when identifying clinically actionable therapeutic alterations comprising variants that could be targeted by a United States Food and Drug Administration (US FDA)-approved or off-label drug, or those that were the target of an ongoing clinical trial. We also determined WfG's ability to detect variants of diagnostic or prognostic significance. Lastly, we performed a comparison of acute myeloid leukemia (AML) oncogenic drivers between South Korean, Caucasian, African American, and Hispanic cohorts using a publicly available database to investigate any differences among these ethnicities.^[9]^

## 2 Materials and methods

### 2.1 Patients

**Figure 1:**
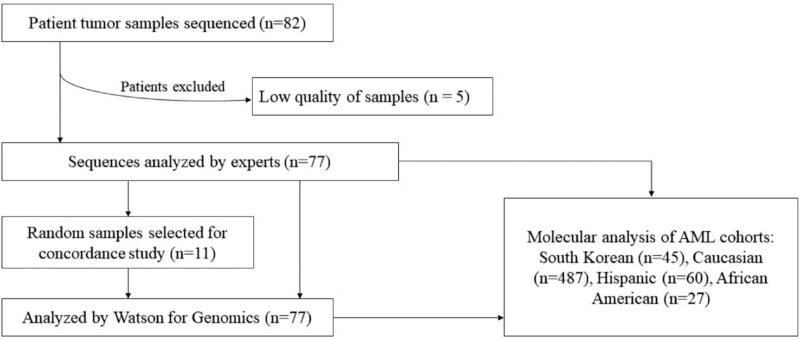
Flow diagram showing the selection of 77 South Korean patients with hematologic malignancies for this study. AML = acute myeloid leukemia.

A flow diagram of the patient selection process is shown in Figure 1. NGS was performed on the bone marrow aspirates of 82 patients diagnosed with hematological malignancies at Hallym University Sacred Heart Hospital, South Korea, between December 2017 and August 2020; good-quality sequencing data were obtained from 77 of them, while the remaining 5 were excluded. The patient characteristics are summarized in Table 1; AML (58%) was the most frequent diagnosis. The study was approved by the Institutional Review Board of Hallym University Sacred Heart Hospital (No.: HALLYM 2018-12-028), and the requirement for written informed consent was waived. The study was performed in accordance with principles of the Declaration of Helsinki.

**Table 1:** Characteristics of the 77 South Korean patients with hematologic malignancies.

Characteristic	Value
Age, mean (range)	62.75 (18–89) yr, unspecified 15
Sex, male/female	33 (42%)/29 (38%), unspecified 15 (20%)
Diagnosis	
Acute myeloid leukemia	45 (58.4%)
Acute myeloid leukemia with recurrent genetic abnormalities	6 (13.3%)^∗^
Acute myeloid leukemia with *RUNX1-RUNX1T1*	1 (16.7%)^†^
Acute myeloid leukemia with *PML-RARA*	2 (33.3%)^†^
Acute myeloid leukemia with *CBFB-MYH11*	3 (50.0%)^†^
Acute myeloid leukemia with myelodysplasia-related changes	5 (11.1%)^∗^
Acute myeloid leukemia, not otherwise specified	34 (75.6%)^∗^
Myeloproliferative neoplasms including chronic myeloid leukemia	12 (15.6%)
Myelodysplastic syndromes	6 (7.8%)
Multiple myeloma	7 (9.1%)
Others	7 (9.1%)

∗ Percentage among acute myeloid leukemia.

† Percentage among acute myeloid leukemia with recurrent genetic abnormalities.

### 2.2 Sequencing workflow

In-house genome sequencing was performed on hematological tumor samples using a 54-gene Illumina TruSight Myeloid panel. Sequencing libraries were prepared and analyzed on a MiSeq sequencer (Illumina, San Diego, CA). Orthogonal testing for *CEBPA* and *FLT3* internal tandem duplications (ITDs) were performed using Sanger sequencing.^[10,11]^ Two other confirmatory tests for the detection of *FLT3*-ITD were performed: *FLT3*-ITD fragment analysis using LeukoStrat (InVivoScribe, San Diego, CA) and *FLT3*-ITD polymerase chain reaction (Biosewoom, Seoul, Republic of Korea).

### 2.3 WfG workflow

**Figure 2:**
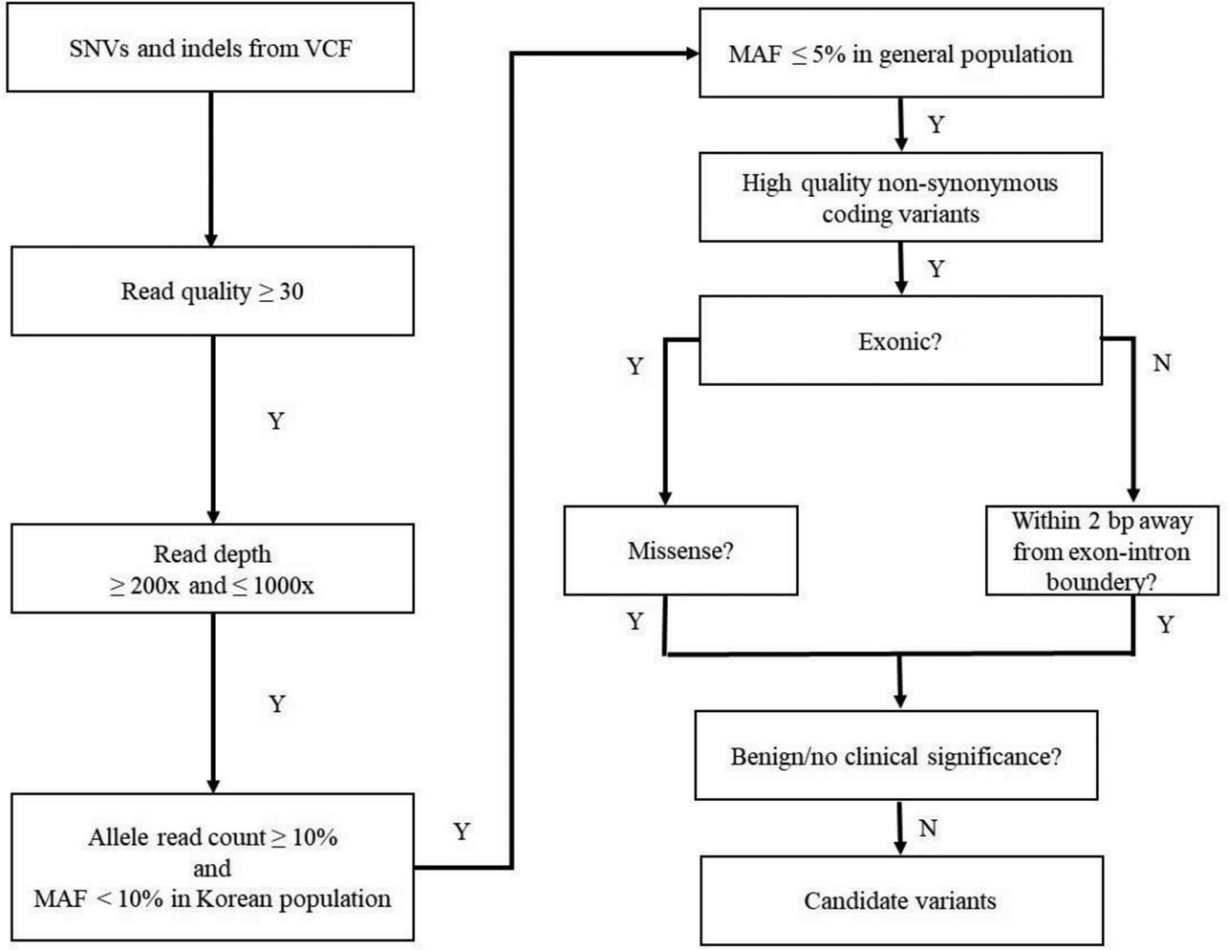
Workflow of candidate variants selection. bp = base pair, MAF = minor allele frequency, SNP = single nucleotide polymorphism, SNV = single nucleotide variant, VCF = variant call format.

Figure 2 illustrates the original variant call format (VCF) filtering process. Variants with a “Phred” quality score >30 were subjected to the downstream analysis. Additional filters were applied to the original VCFs to obtain high-quality candidate mutation variants, resulting in the exclusion of those with read depths of <200× or allele frequencies under 10%, those with read depths outside the range of 200× to 1000×, and those with common single nucleotide polymorphisms (i.e., minor allele frequencies present in >5% of the general population or in >10% of the South Korean population).

After removing low-quality variants, synonymous and noncoding variants plus well-documented single nucleotide polymorphisms that are known to be benign, likely benign, or of no clinical significance were further filtered out. At the splicing sites, variants detected within 2 base pairs from exon-intron boundaries were included for downstream analysis. The selected candidate variants were then analyzed using WfG version 49.

WfG was used for variant interpretation and annotation of all the patients’ sequencing results, which were then compared to manual curation performed by experts. Each patient's cancer type and list of variants were uploaded to WfG as a VCF file. The report provided by WfG included (a) variants categorized according to the degree of pathogenicity (pathogenic, likely pathogenic, and variant of unknown significance [VUS]); (b) a list of therapeutic options that included US FDA-approved drugs and currently recruiting clinical trials categorized by level of evidence; and (c) resistance information (when applicable). The WfG report contained these results as well as supporting evidence extracted from peer-reviewed publications, selected databases, and clinical trials.

### 2.4 Gene mutation comparison and statistical analysis

Forty-five AML samples were used to explore the differences in mutational signatures between patients in our South Korean cohort and those of Caucasian, African American, and Hispanic descent from a previously published study.^[9]^ Statistical tests were run to identify clinically actionable biomarkers that significantly varied between the South Korean (n = 45), Caucasian (n = 487), Hispanic (n = 60), and African American (n = 27) cohorts. The frequency of variants was compared using Fisher's exact test, with a *P* value of <.05 considered statistically significant without correcting for multiple comparisons.

## 3 Results

### 3.1 Gene mutation concordance between expert panel findings and WfG

The interpretations of NGS data by WfG and the expert panel were compared in 11 randomly selected cases (10 AML and 1 primary myelofibrosis, male:female ratio = 7:4) to determine the accuracy of WfG. The gene variants (see Table S1, http://links.lww.com/MD2/A789 Supplemental Content, which lists these variants) were 94% concordant (33/35). *ASXL1* c.1934dupG p.Gly646fs variants in all cases were excluded owing to an “alignment error/artifact” classification. Twenty-seven of the 35 mutations (77%) were identified as pathogenic or likely pathogenic by both interpretations (See Table S2, http://links.lww.com/MD2/A790 Supplemental Content, for comparisons between WfG- and manual curation-derived annotations). In the VUS subgroup, 6 of 35 mutations (17%) were identified using both methods. WfG interpreted 1 gene mutation (3%) that was classified as VUS by manual interpretation as “likely pathogenic” and did not detect another mutation (3%) that was also manually determined to be VUS.

### 3.2 Clinically actionable therapeutic alterations reported by WfG

According to WfG, 51% of all patients (39/77) had at least 1 clinically actionable therapeutic alteration (i.e., a variant targeted by a US FDA-approved drug, off-label drug, or clinical trial); 46% of these cases (18/39) had gene variants that were targeted by a US FDA-approved therapy. Specifically, 7 patients had *IDH1* and *IDH2* mutations that rendered them eligible for the US FDA-approved agents ivosidenib and enasidenib, respectively. Four patients who had loss-of-function mutations in *DNMT3A* could potentially derive a clinical benefit from histone deacetylase inhibitors and other methylation regulators.^[12]^ Six patients who had *JAK2* mutations could therefore be candidates for *JAK* inhibitors. Finally, 2 patients had pathogenic *SF3B1* mutations that allowed them to be potential candidates for certain ongoing AML clinical trials.

WfG identified diagnostic or prognostic insights in 24 of the 37 patients (65%) who had no therapeutic alterations, while the remaining 14 had no clinically actionable therapeutic, diagnostic, or prognostic information. Twelve patients with *NPM1* mutations were identified, although they did not harbor *FLT3*-ITD mutations that have been associated with favorable prognoses in AML^[13]^; 1 of these 12 patients had a concomitant *FLT3* tyrosine kinase domain mutation, which has been associated with good prognosis.^[14]^ One patient had a *CEBPA* mutation (confirmed by both NGS and Sanger sequencing) that has been associated with favorable outcomes.^[15]^

### 3.3 Comparison of AML markers in Koreans versus other ethnicities

A comparison of key biomarkers present in South Korean patients with AML versus those present in Caucasian, Hispanic, and African American counterparts as described in a previous study^[9]^ was performed (Table 2). *FLT3*-ITD or tyrosine kinase domain mutations were reported in 30% of the patients in the Caucasian cohort (147/487) but were significantly lower at 6.7% (3/45) among those in the South Korean cohort (*P* = <.001). When comparing the South Korean cohort with only the Hispanic patients (n = 60), the difference in *FLT3* variant frequencies was also significant (*P* = .005); however, no significant differences were identified for the other biomarkers. The differences in the frequencies of other common AML biomarkers (including *NPM1*, *DNMT3A, IDH1, IDH2, TP53, KRAS, NRAS, RUNX1,* and *CEBPA*) between the South Korean and other cohorts were not significant. Finally, no significant differences in variant frequencies were observed between the South Korean and African American (n = 27) cohorts; notably, the prevalence of *FLT3* mutations in both these cohorts was similar.

**Table 2:** Comparison of alterations in key genes detected in patients with acute myeloid leukemia from different ethnicities.

	South Korean	Caucasian		Hispanic		African American	
Genes	n = 45	n = 487	*P* value^∗^	n = 60	*P* value^∗^	n = 27	*P* value^∗^
*FLT3*	3 (6.7%)	147 (30.2%)	.0004	17 (28.3%)	.005	4 (14.8%)	.413
*NPM1*	12 (27%)	116 (23.8%)	.72	11 (18.3%)	.346	3 (11.1%)	.139
*DNMT3A*	14 (31.1%)	119 (24.4%)	.36	9 (15.0%)	.058	6 (22.2%)	.587
*IDH1*	3 (6.7%)	42 (8.6%)	1.000	3 (5.0%)	1.000	6 (22.2%)	.071
*IDH2*	6 (13.3%)	63 (12.9%)	1.000	7 (11.7%)	1.000	4 (14.8%)	1.000
*TP53*	6 (13.3%)	39 (8.0%)	.256	4 (6.7%)	.320	7 (25.9%)	.214
*KRAS*	1 (2.2%)	17 (3.5%)	1.000	6 (10.0%)	.234	1 (3.7%)	1.000
*NRAS*	5 (11.1%)	61 (12.5%)	1.000	11 (18.3%)	.413	1 (3.7%)	.399
*RUNX1*	7 (15.6%)	59 (12.1%)	.480	9 (15.0%)	1.000	3 (11.1%)	.733
*CEBPA*	2 (4.4%)	29 (6.0%)	1.000	2 (3.3%)	1.000	0 (0.0%)	.524

∗ *P* values were calculated using Fisher's exact test, which was used to compare the incidences in the South Korean cohort to those in each of the 3 other ethnic cohorts.^[9]^

## 4 Discussion

Our results showed that the variant interpretations for hematological tumors by WfG correlated well with those manually curated by experts, thereby validating the accuracy of the former in patients with these types of malignancies. WfG identified clinically actionable therapeutic alterations, as well as variants with diagnostic or prognostic insights, in a substantial proportion of the examined cases. Moreover, we found that the frequency of *FLT3*-ITD in South Koreans with AML was lower than that in Caucasian and Hispanic counterparts.

The variant identification and annotation concordance rate between expert-performed manual curation (the standard method) and WfG was over 94%, demonstrating the competency of WfG in hematologic malignancies for the first time. WfG has obviated the need for labor-intensive manual curation of clinical trials and therapies, enabling our center to exponentially scale up our NGS operations. As was also reported by investigators at the University of North Carolina, WfG was able to identify nearly all variants previously defined as actionable by the molecular tumor board in <3 minutes per case; such analyses would typically take hours or days to perform manually.^[16]^ These results demonstrate that the use of technology to support the accurate interpretation of somatic NGS results may greatly enhance the ability of clinicians to make informed decisions at the point of care. Molecular tumor boards empowered by artificial intelligence may be able to improve patient care by providing a rapid, comprehensive, and high-quality approach to data analysis and presenting up-to-date information on available clinical trials.

Genomic sequencing of solid tumors provides insights into targeted therapeutic interventions and alterations that confer resistance to therapies. Our data confirmed that WfG can contribute to personalized care for patients with hematologic malignancies given that it identified 51% of all patients as having at least 1 clinically actionable therapeutic alteration. In particular, 46% of patients had gene variants (including *JAK2, IDH1, IDH2*, and *DNMT3A*) that rendered them eligible for a US FDA-approved therapy. In 65% of cases without therapeutic alterations, WfG identified variants of diagnostic or prognostic significance including *NPM1, FLT3*, and *CEBPA*, which assisted clinicians in arriving at the correct diagnoses and/or facilitated risk-stratification for individual patients.

An evidence-based artificial intelligence solution can also facilitate the interrogation of ethnic disparities in oncogenic molecular profiles. In our study, the frequencies of *FLT3* mutations were lower in South Koreans than in Caucasians and Hispanics, suggesting that some mutational signatures that predict cancer outcomes may vary by race. *FLT3* mutations are associated with poor prognoses, increased relapse rates, and decreased overall survival.^[17]^*FLT3* mutations were less frequent in South Korean patients with AML (6.7%) than in counterparts from other ethnic groups (generally reported to be 23%–27%).^[18,19]^ A previous study also found the prevalence of this mutation among South Koreans to be relatively low (13%).^[20]^ Interestingly, we did not detect the *FLT3* D835 kinase domain-activating mutation in the South Korean cohort. The low prevalence of *FLT3* mutations in South Koreans suggests that caution should be exercised when prescribing FLT3 inhibitors such as midostaurin and gilteritinib to these patients before their tumors have been sequenced. While not significant, *TP53* mutations were observed with a higher frequency in Koreans compared to Caucasians and Hispanics. *TP53* missense mutations result in a loss of protein function and exert a dominant negative effect in AML^[21]^; such mutations have also been associated with low responses to chemotherapy and poor prognoses.^[22]^ However, patients with AML who harbor *TP53* mutations have responded favorably to decitabine^[23]^; moreover, *TP53* mutations are reportedly useful as a risk stratification parameter.^[24]^ Therefore, the higher prevalence of *TP53* mutations among South Korean patients with AML may have important therapeutic and prognostic implications.

A limitation of our study was the small cohort size. Additional key genes with frequencies that are significantly different from those in patients of other ethnicities or even between Korean patients with different AML subtypes may have been discovered if our study had comprised a larger cohort of South Korean patients with hematologic malignancies.

In summary, ours was the first study to investigate the performance of an evidence-based artificial intelligence solution, WfG, in hematologic malignancies in terms of (1) concordance with expert-performed manual variant annotations/interpretations, and (2) identification of druggable targets or variants harboring other features of clinical significance. Our results ought to serve as a springboard for further studies with larger cohorts and more diverse types of hematologic malignancies. WfG results were highly concordant with those of expert manual curation, and provided diagnostic, prognostic, and/or therapeutic insights for a majority of patients within a few minutes. Our study showed that evidence-based artificial intelligence solutions such as WfG are fast and accurate enough to contribute to the timely management of patients with hematologic malignancies. This, in turn, allows the incorporation of high-throughput molecular profiling into routine practice, thereby moving precision oncology a step forward. Additionally, the differences in the frequencies of *FLT3* and *TP53* mutations between South Koreans and Caucasians with AML suggest ethnic differences in the pathogenesis of this disease, which may lead to the additional stratification of this type of malignancy.

## Acknowledgments

The authors gratefully acknowledge the support of Eunyup Lee, Jee-Soo Lee, Chinta Bagwe, Irene Dankwa-Mullen, Winnie Felix, and Lionel Lim.

## Author contributions

**Conceptualization:** Jane L Snowdon, Dilhan Weeraratne, Hu Huang, Miyoung Kim.

**Data curation:** Jane L Snowdon, Dilhan Weeraratne, Shang Xue, Young Kyung Lee, Kibum Jeon, Dae Young Zang, Hyo Jung Kim, Ho Young Kim, Boram Han, Miyoung Kim.

**Formal analysis:** Jane L Snowdon, Dilhan Weeraratne, Hu Huang, Shang Xue, Miyoung Kim.

**Writing – original draft:** Jane L Snowdon, Dilhan Weeraratne, Hu Huang, David Brotman, Van C Willis, Miyoung Kim.

**Writing – review & editing:** David Brotman, Van C Willis, Miyoung Kim.
